# Variable Selection and Joint Estimation of Mean and Covariance Models with an Application to eQTL Data

**DOI:** 10.1155/2018/4626307

**Published:** 2018-06-25

**Authors:** JungJun Lee, SungHwan Kim, Jae-Hwan Jhong, Ja-Yong Koo

**Affiliations:** ^1^Department of Statistics, Korea University, Seoul 02841, Republic of Korea; ^2^Department of Applied Statistics, Konkuk University, Seoul 05029, Republic of Korea

## Abstract

In genomic data analysis, it is commonplace that underlying regulatory relationship over multiple genes is hardly ascertained due to unknown genetic complexity and epigenetic regulations. In this paper, we consider a joint mean and constant covariance model (JMCCM) that elucidates conditional dependent structures of genes with controlling for potential genotype perturbations. To this end, the modified Cholesky decomposition is utilized to parametrize entries of a precision matrix. The JMCCM maximizes the likelihood function to estimate parameters involved in the model. We also develop a variable selection algorithm that selects explanatory variables and Cholesky factors by exploiting the combination of the GCV and BIC as benchmarks, together with Rao and Wald statistics. Importantly, we notice that sparse estimation of a precision matrix (or equivalently gene network) is effectively achieved via the proposed variable selection scheme and contributes to exploring significant hub genes shown to be concordant to* a priori* biological evidence. In simulation studies, we confirm that our model selection efficiently identifies the true underlying networks. With an application to miRNA and SNPs data from yeast (a.k.a. eQTL data), we demonstrate that constructed gene networks reproduce validated biological and clinical knowledge with regard to various pathways including the cell cycle pathway.

## 1. Introduction

Generally, joint estimation of mean and covariance has been developed to address problems related to biomedical data. In longitudinal data analysis, for instance, identifying correct correlation structures within each subject is a major focus and many studies come up with variants of joint mean and covariance estimation to enhance statistical efficiency (see Ye and Pan [1] and references therein). With regard to graphical models, particularly, conditional Gaussian graphical models (cGGM) aiming to elucidate conditional dependence structures subject to the mean have increasingly received much attention [2–5] and conceptually can also be viewed as examples of joint mean and covariance estimation in essence. On a methodological side, joint estimation has been found to be practically applicable in the sense that applications of joint estimation methods cover bioinformatics, such as hormone and transcriptome [1, 4, 6–8], and stock price analysis [9]. A majority of existing methods involve variable selection of covariates for the mean vector and sparsity of covariance mainly based on the penalization methods. However, in this paper, we purposely focus on variable selection and joint estimation with a pure implementation of the likelihood-based method, unlike common penalization techniques, to better analyze eQTL data in light of genetic feature selection.

Over the decades, genomics research has focused on comprehensive understanding of regulatory networks in the context of system biology. Commonly we are interested in a gene network, which pictures the interplay among genetic factors (e.g., gene regulation and activation). Particularly, it is important to investigate how a given genotype (genetic variants) at a particular quantitative trait locus (QTL) affects measured phenotypes and traits at that locus. For instance, gene expression quantitative loci (eQTL) make use of gene expressions as quantitative traits. eQTL analysis has been widely applied to figure out the effect of genetic perturbations associated with diseases as well as to construct regulatory networks describing how genes regulate expressions of other genes [10, 11]. More precisely, the location of single nucleotide polymorphisms (SNPs) may affect multiple gene expression levels, and this accidentally causes misleading inference for dependency structure among genes [4]. Many popular methods [2–5, 12] have been introduced to identify gene networks, aiming at learning networks subject to perturbation effects by genetic variants on the basis of population gene expression and genotype data.

Yin and Li [4] proposed the sparse conditional graphical Gaussian model with *ℓ*_1_ and the adaptive lasso penalty function. Li* et al.* [3] suggested the two-stage estimation framework: (1) estimating a nonsparse conditional covariance matrix of genes based on a conditional variance operator between the reproducing kernel Hilbert spaces of marker genes and then (2) using *l*_1_ and adaptive lasso penalty to obtain sparse estimates of a precision matrix under the cGGM. Cai* et al.* [12] studied the covariate-adjusted precision matrix estimation (CAPME) method using the constrained *ℓ*_1_ optimization.

While most recent studies encourage sparsity estimation of a precision matrix based on the penalized likelihood, we instead rely on classical variable methods based on the standard likelihood. Strictly speaking, the penalized likelihood, according to its definition, cannot be viewed as the likelihood. It naturally poses a question whether the likelihood-based method performs better than the penalized likelihood approaches. To address this question, we consider a joint mean and constant covariance model (JMCCM) inspired by Pourahmadi [13] and propose methods for variable selection which effectively identify sparse conditional gene networks and covariates relevant to gene regulations. We employ the modified Cholesky decomposition to guarantee positive definiteness of an estimated precision matrix [13]. With this reparametrization of the precision matrix, the log-likelihood function corresponding to our model can be decomposed into an additive form of each response in terms of Cholesky parameters. This facilitates a coordinate descent type implementation we adopt for the precision matrix estimation. The combination of both generalized cross-validation (GCV) and Bayesian information criterion (BIC) performs variable selection as a benchmark. Rao and Wald statistics are also utilized to add and delete genetic markers for each gene expression and the Cholesky factors for the precision matrix. To the best of our knowledge, only a few works use Rao and Wald statistics for variable selection in the joint estimation problem, particularly with an application to eQTL analysis.

In simulation studies, extensive simulation scenarios experimentally confirm improved estimation efficiency and precise variable selection of the proposed method. For real data applications, we perform eQTL analysis via the JMCCM with gene expressions and SNPs of yeast data, in pursuit of detecting the effects of variant perturbations and underlying gene regulations. We find that the JMCCM effectively uncovers biological pathways that may potentially account for known biological processes. Taken together, the JMCCM is shown to be effective in identifying conditional dependence structures among variables compared to the existing graphical models with penalization methods such as the sparse cGGM [5] and CAPME [12].

The paper is outlined as follows. In Section 2, we describe our JMCCM with modified Cholesky decomposition and maximum likelihood estimates. The variable selection algorithm using Rao and Wald statistics for SNPs and Cholesky factors is explained in Section 3.  Section 4 deals with simulation studies to demonstrate performance of variable selection and estimation of the proposed model. The yeast cell cycle pathway genes with SNPs data analysis are presented in Section 5. Concluding remarks and discussion in Section 6 are followed by the Appendix, which provides mathematical details of our method.

## 2. Model And Estimators

### 2.1. JMCCM with the Modified Cholesky Decomposition

Contrary to previous methods [3, 4], the JMCCM primarily aims at simultaneous estimation over the mean and precision matrix of the Gaussian graphical model. Suppose that (*x*, *y*) is a pair of the *p* × 1 vector *x* of genetic markers and the *m* × 1 random vector *y* of expression levels. Let *x* denote the vector of SNPs and let *y* denote the gene expression traits following *m*-variate multivariate normal distribution with the mean *μ*(*x*; *β*) and covariance matrix Σ as follows:

(1) $$\begin{array}{c} y\;|\;x\sim N_{m}\left(\;\mu \left(\;x;\beta \;\right),\Sigma \;\right), \end{array}$$

where the *j*th entry of *μ*(*x*; *β*) is *x*^*⊤*^*β*_*j*_ for *j* = 1,…, *m*, and *β*_*j*_ = (*β*_*j*_^1^,…, *β*_*j*_^*p*^) is the *p* × 1 linear regression coefficient vector indicating effects of SNPs perturbations to gene expressions and *β* = (*β*_1_,…, *β*_*m*_). Importantly, note that Σ does not depend on *x*. The coefficient *β* is assumed to be sparse, since each gene is known to have only a few genetic regulators according to Cai* et al.* [12]. The precision matrix represents a gene network (graph), as in an undirected GGM, by corresponding the nonzero (*i*, *j*)th element of the precision matrix with an edge between two vertices *i* and *j* [14]. This edge represents conditional dependence of genes *i* and *j* given all other gene expression levels. Thus, our goal is to identify nonzero entries of the precision matrix in order to construct a conditional dependence genetic network after the effects of SNPs perturbations are removed. The precision matrix Σ^−1^ is also expected to be sparse [4].

One of our primary interests is to estimate the precision matrix Σ^−1^, which is symmetrically positive definite, so we need to ensure that the estimate of the precision matrix also satisfies symmetrical positive definiteness. To this end, we apply the modified Cholesky decomposition [13] to the precision matrix, denoted by *K*, as follows:

(2) $$\begin{array}{c} K\left(\;\phi ,\tau \;\right)=C{\left(\;\phi \;\right)}^{⊤}D\left(\;\tau \;\right)C\left(\;\phi \;\right), \end{array}$$

where *C* is an upper triangular matrix with diagonal entries 1 and above-diagonal elements consisting of negative of *ϕ* = (*ϕ*_12_,…, *ϕ*_1*m*_, *ϕ*_23_,…, *ϕ*_2*m*_,…, *ϕ*_*m*−1,*m*_) and *D* is a diagonal matrix containing *τ* = (*τ*_1_,…, *τ*_*m*_) with *τ*_*j*_ > 0 for all *j* = 1,…, *m* as diagonal entries. Here, the superscript "*⊤*" denotes the transpose of a matrix. Positive definiteness of *K* is shown in Appendix A. Throughout this paper, a vector in the parenthesis is considered as a column vector. Let *ϕ*_*j*_ = (*ϕ*_*j*,*j*+1_,…, *ϕ*_*jm*_) for *j* = 1,…, *m* − 1. Then we can write *ϕ* = (*ϕ*_1_,…, *ϕ*_*m*−1_). The parameter space for JMCCM is defined by Θ = {*θ* = (*β*, *ϕ*, *τ*) : *β* ∈ *ℝ*^*mp*^, *ϕ* ∈ *ℝ*^*m*(*m*−1)/2^  and  *τ* ∈ *ℝ*_+_^*m*^}, where *ℝ*_+_^*m*^ represents the set of *m*-dimensional vectors of positive real numbers.

### 2.2. Maximum Likelihood Estimation

Suppose that we have the *N* independent observations (*x*^*i*^, *y*^*i*^), *i* = 1,…, *N*, sampled from (1), where *y*^*i*^ = (*y*_1_^*i*^,…, *y*_*m*_^*i*^) and *x*^*i*^ = (*x*_1_^*i*^,…, *x*_*p*_^*i*^) represent the *i*th observation of *x* and *y*, respectively. With Σ^−1^ = *K* = *K*(*ϕ*, *τ*), *μ*(*x*) = *μ*(*x*; *β*), and *μ*^*i*^ = *μ*(*x*^*i*^), the log-likelihood function corresponding to (1) is given by

(3) $$\begin{array}{c} l\left(\;\theta \;\right)=\frac{N}{2}\left\{\;-m\log\left(\;2\pi \;\right)+\overset{m}{\underset{j=1}{\sum }}\log\tau_{j}-tr\left(\;KV\left(\;\beta \;\right)\;\right)\;\right\}, \end{array}$$

where *V*(*β*) = (1/*N*)∑_*i*=1_^*N*^(*y*^*i*^ − *μ*^*i*^)(*y*^*i*^ − *μ*^*i*^)^*⊤*^ and "tr" denotes the trace of a matrix. A derivation of (3) can be found in Appendix B. The maximum likelihood estimator (MLE) of *θ* is defined by

(4) $$\begin{array}{c} \hat{\theta }=\underset{\theta \in \Theta }{\mathrm{argmax}}l\left(\;\theta \;\right). \end{array}$$

Some notations are needed to express MLE of *θ* in conjunction with variable selection. For *j* = 1,…, *m*, let *G*_*j*_ be an index set of active (or significant) coefficients of *β*_*j*_ and denote by *g*_*j*_ the number of elements in *G*_*j*_. Let *G* = {*G*_1_,…, *G*_*m*_} and *g* = *g*_1_ + ⋯+*g*_*m*_. Obviously, *g* ≤ *mp* and if *g* = *mp*, then all variables are significant for all *y*_1_,…, *y*_*m*_. Define *β*(*G*) = (*β*_1_(*G*_1_),…, *β*_*m*_(*G*_*m*_)) ∈ *ℝ*^*g*^ and $\beta_{j}(G_{j})={\left[\begin{array}{c} \beta_{j}^{l} \end{array}\right]}_{l\in G_{j}}\in {\mathbb{R}}^{g_{j}}$ for *j* = 1,…, *m*. Write *y*_*k*_ = (*y*_*k*_^1^,…, *y*_*k*_^*N*^) for *k* = 1,…, *m*, *X* = (1/*N*)(*x*_1_,…, *x*_*p*_), and *X*(*G*_*j*_) = [*X*_*l*_]_*l*∈*G*_*j*__ ∈ *ℝ*^*N*×*g*_*j*_^, where *X*_*l*_ denotes *l*th column of *X*. Let *Y* = (*y*_1_^*⊤*^,…, *y*_*m*_^*⊤*^) ∈ *ℝ*^*mN*^, *X* = ⊕_*j*=1_^*m*^*X*(*G*_*j*_) ∈ *ℝ*^*mN*×*g*^, and *W* = *K* ⊗ *I*_*N*_ ∈ *ℝ*^*mN*×*mN*^, where ⊕ and ⊗ represent direct sum and Kronecker product, respectively, and *I*_*N*_ is the *N* × *N* identity matrix. Then the MLE $\hat{\beta }(G)$ of *β*(*G*) can be expressed as

(5) $$\begin{array}{c} \hat{\beta }\left(\;G\;\right)={\left(\;X^{⊤}WX\;\right)}^{-1}X^{⊤}WY. \end{array}$$

Observe that estimating *β*(*G*) involves *K*.

We can estimate *K* by obtaining MLE of *ϕ* and *τ*. For *j* = 1,…, *m* − 1, let *θ*_*j*_ = (*β*, *ϕ*_*j*_, *τ*_*j*_) with *θ*_*m*_ = (*β*, *τ*_*m*_). Let *V*_*jj*_, *V*_*j*,21_, and *V*_*j*,22_ represent the (1,1), (2,1), and (2,2) components of the lower *j*th principal submatrix *V*_*j*_(*β*) of *V*(*β*). Then we can express

(6) $$\begin{array}{c} l\left(\;\theta \;\right)=\overset{m}{\underset{j=1}{\sum }}l_{j}\left(\;\theta_{j}\;\right), \end{array}$$

where

(7) ljθj=N2−log2π+log⁡τj−τjVjj−2ϕj⊤Vj,21+ϕj⊤Vj,22ϕj.

By (6), we can obtain $\left(\hat{\phi },\hat{\tau }\right)$ from $\left({\hat{\phi }}_{j},{\hat{\tau }}_{j}\right)$ for each *j* with ${\hat{\phi }}_{j}=argmax_{\phi_{j}}\ell_{j}(\theta_{j})$ and ${\hat{\tau }}_{j}=argmax_{\tau_{j}}\ell_{j}(\theta_{j}).$ Thus, instead of finding a solution to the optimization problem with (3), we optimize each *ℓ*_*j*_(*θ*_*j*_) with respect to *ϕ*_*j*_ and *τ*_*j*_. Thus, the MLE ${\hat{\phi }}_{j}$ and ${\hat{\tau }}_{j}$ are expressed in terms of *V*(*β*) in a way that

(8) ϕ^j=Vj,22−1Vj,21,1τ^j=Vjj−Vj,12Vj,22−1Vj,21for  j=1…,m−1

with $\hat{\beta }$ from (5) and $1/{\hat{\tau }}_{m}=V_{mm}$. We estimate Σ^−1^ by $\hat{K}$ and it is defined by

(9) $$\begin{array}{c} \hat{K}=K\left(\;\hat{\phi },\hat{\tau }\;\right). \end{array}$$

Derivations of (5), (6), and (8) are presented in Appendix C.

## 3. Consecutive Variable Selection Algorithm

As mentioned above, *β* and *K* are commonly believed to be sparse in genomic data analysis. To address sparsity, the lasso-type penalty imposed on both regression coefficients and precision matrix has been popularly applied to diverse graphical models [4, 5]. Stepping aside the lasso-type approach, we develop a variable selection technique that mainly relies on the combination of classical variable selection methods. Generally, the numbers of SNPs and genes tend to be considerably huge so that computational costs normally become prohibitive. In order to address this problem, the proposed variable selection algorithm proceeds with largely two stages: (1) preliminary variable selection for mean and precision matrix and (2) secondary variable selection in the middle of the joint model estimation. It is important to note that the first stage leads possible variables to be limited in scope (i.e., working parameters in the model) in order to circumvent high computational complexity.

### 3.1. Preliminary Variable Selection

Preliminary variable selection is largely twofold: variable selection for the mean part and covariance part. The idea behind that is to add variables (or equivalently parameters) to the joint model with the maximum Rao statistic and to delete ones with the minimum Wald statistic. You may refer to Koo [15] for the basis selection method or Kooperberg* et al.* [16] that explain variable selection schemes based on Rao and Wald statistics.

#### 3.1.1. Mean Part

When it comes to the mean part, we carry out selecting predictor variables (i.e., SNPs) for each response variable (i.e., gene expression), dealing with a univariate multiple regression problem. In the addition stage, we start off with a model including only an intercept term. The MLE is used as estimator for *β* and maximum Rao statistic (Rao [17]) is the criterion for adding a predictor together with GCV (Friedman [18]) as a stopping rule. In the deletion stage, Wald statistic is calculated to exclude predictor variables such that the updated model minimizes Wald statistics. The final model for the mean regression is chosen by the minimum GCV. Details are summarized in Algorithm 1. Once Algorithm 1 is done, the number of predictor variables included in the joint model no longer increases, while variable reduction can happen in Algorithm 3 (Section 3.2).

#### 3.1.2. Covariance Part

The rationale behind variable selection in the precision matrix estimation is that each *Y*_*j*_, *j* = 1,…, *m* − 1, is regressed on *Y*_*j*+1_,…, *Y*_*m*_, and the regression coefficients are negative of off-diagonal entries of *C*(*ϕ*), the by-product of the modified Cholesky decomposition (2) [13, 19, 20]. Clearly, this is one of the major benefits of reparametrization via Cholesky decomposition [13] in pursuit of improved interpretation.

Subsequent to selection for predictor variables, we compute $V(\hat{\beta }(G))$. Given initial ${\hat{\phi }}_{j}^{\left(0\right)}=0$, we start with computing ${\hat{\tau }}_{j}$ for *j* = 1,…, *m* as in (8) using $V(\hat{\beta }(G))$ obtained from Algorithm 1. Then compute Rao statistic to add one variable out of *Y*_*j*+1_ …, *Y*_*m*_ to the current model that builds on the response variable *Y*_*j*_. We repeatedly update for *Y*_*j*_, where *j* = 1,…, *m* − 1. Next, we choose one variable, say *ϕ*_*lb*_, for *l* = 1,…, *m* − 1 and *b* = *l* + 1,…, *m*, such that Rao statistic is maximized and thereby update ${\hat{\phi }}_{l}$ and ${\hat{\tau }}_{l}$, respectively. When calculating $\hat{\tau }$ and $\hat{\phi }$, the BIC is computed, and the addition process stops if the BIC no longer decreases. At the completion of addition, we build a model with all selected variables as a full model and subsequently begin deletion from a full model. Deletion process is similar to the addition process except that the minimum Wald statistic is used for deletion in place of the maximum Rao statistic. Successive deletion continues until the BIC stops decreasing. In the last stage, the final model is also selected by the BIC. Details are presented in Algorithm 2. Once Algorithm 2 is finished, the number of *ϕ*_*jk*_ included in the joint model no longer increases, while variable reduction could occur in the joint estimation (Algorithm 3, Section 3.2). Sparsity of $\hat{K}$ is achieved through this variable selection scheme along with Algorithm 3.

### 3.2. Secondary Variable Selection in the Middle of the Joint Model Estimation

With variables (i.e., parameters) selected by the preliminary stage above, we implement simultaneous parameter estimation for both mean and precision in the joint model and additional variable selection. Once $\hat{K}$ is computed via Algorithm 2 with fixed $\hat{\beta }(G)$, we start joint estimation by updating $\hat{\beta }(G)$, which is formed with weight least square estimates as in (5) and weights coming from $\hat{K}$. Then $\hat{\phi }$ and $\hat{\tau }$, ultimately $\hat{K}$, are newly computed using updated $V(\hat{\beta }(G))$, and again this updated $\hat{K}$ serves as a weight for updating $\hat{\beta }(G)$. Over the updates, the interplay between $V(\hat{\beta }(G))$ and $\hat{K}$ continues until the log-likelihood function converges. Afterwards, deletion to current parameters begins by Wald statistic, excluding one parameter with the minimum Wald statistic. Finally, the BIC is used as a stopping rule for deletion and selection to finalize model estimation. Algorithm 3 contains details about this procedure. While the preliminary variable selection works for each mean and covariance part under the assumption of $\hat{\beta }(G)$ or $\hat{K}$ are fixed, this joint estimation procedure is designed to improve estimation and selection accuracy by reflecting the changes of $\hat{\beta }(G)$ and $\hat{K}$.

## 4. Experimental Studies

In order to assess the performance of our proposed method, we carry out experimental simulations and compare the sparse conditional Gaussian graphical model (SCGGM), Zhang and Kim [5], covariate-adjusted precision matrix estimation (CAPME), Cai* et al.* [12] and joint model with lasso penalty (JML), and Jhong* et al.* [21], all of which are based on penalized likelihood approaches. The competing methods are run with their default setting regarding tuning parameters. To evaluate similarity, the estimated precision matrix and true matrix are benchmarked by the Steins loss function:

(10) $$\begin{array}{c} \delta_{Stein}\left(\;K,\hat{K}\;\right)=tr\left(\;K{\hat{K}}^{-1}\;\right)-log\left|\;K{\hat{K}}^{-1}\;\right|-m, \end{array}$$

where $\hat{K}$ is an estimate of the true precision matrix *K* and |·| denotes the determinant of a matrix. The Frobenius norm of difference between *K* and $\hat{K}$, denoted by ‖Δ‖_*F*_, where $\Delta =K-\hat{K}$, is also considered. In addition to the Steins loss function, to measure how efficiently our model recovers the true conditional dependent relationship among genes, specificity (SPE) and sensitivity (SEN) are used, as defined by

(11) SPE=TNTN+FP,SEN=TPTP+FN,

where TN, TP, FN, and FP are the numbers of true negatives, true positives, false negatives, and false positives with regard to off-diagonal elements of a precision matrix. Here, we treat a nonzero entry of a precision matrix as “positive.” To combine sensitivity and specificity, Youden's index (=SPE + SEN − 1) is used. The smaller values of *δ*_*Stein*_ and ‖Δ‖_*F*_ are better, whereas the larger values of SPE, SEN, and Youden are better.

Inspired by Yin and Li [4], we generate simulation data sets in the form of eQTL data sets such that nonzero entries of a precision matrix, commonly called a link (or edge), are randomly assigned with probability *c*_1_/*m*, where *m* is the number of genes and *c*_1_ is some positive constant. For a link generated at the (*i*, *j*)th entry of the precision matrix, denoted by *k*_*ij*_, the corresponding element is sampled from the uniform distribution over [−1,0.5]∪[0.5,1]. For each row, off-diagonal elements are divided by the sum of their absolute values multiplied by 1.5. And we obtain the true precision matrix *K* by symmetrizing and setting diagonals as 1. To create the *p* × *m* regression coefficients matrix *β*, we first generate a *p* × *m* indicator matrix that has 1 as entry with probability *c*_2_/*p* for some positive constant *c*_2_. If the (*l*, *j*)th element of this indicator matrix is 1, *β*_*j*_^*l*^ is randomly generated from *Unif*([*d*_*m*_, 1]∪[−1, −*d*_*m*_]), where *d*_*m*_ is the smallest absolute value of *K* generated.

Producing *β* and *K*, we generate SNPs, *X* = (*X*_1_,…, *X*_*p*_) with *X*_*l*_ ~ Bernoulli(1,0.5) for *l* = 1,…, *p*. Finally, we simulated gene expressions by generating *y* from the multivariate normal distribution given *x*, *Y*∣*X* ~ *N*(*Xβ*, *K*^−1^). We generate a data set of *N* i.i.d. random vectors (*X*, *Y*), and simulations are repeated 50 times. In Table 1, we outline the six simulation scenarios of small-scale setup.  Table 2 contains the three simulation scenarios of large-scale setup.

The small-scale simulation results in Table 3 suggest that the JMCCM produces better estimates than all other methods across all six models. Due to computational issues, we drop some results of JML from Table 3. By comparing the results of model 3 with 6 and 2 with 4, when *N* = 1000 and the number of genes is fixed, we can see that JMCCM and CAPME show less changes in *δ*_*Stein*_ and ‖Δ‖_*F*_ than they appear in the SCGGM as the number of SNPs increases. This indicates that JMCCM and CAPME are less subject to the increment of the number of SNPs than SCGGM, when modest size of genes is involved. Estimation performance of CAPME seems to be affected by the number of genes more easily than JMCCM because Stein loss and Frobenius norm of CAPME for models 1, 2, and 3 with fixed *p* increase more rapidly than those of JMCCM do. For identifying structures of the precision matrix, JMCCM surpasses SCGGM in discovering nonzero elements (higher sensitivity) as complexity of the model rises. This is possibly due to the fact that SCGGM tends to produce sparse estimates more than the true precision matrix. Higher sensitivity implies that our proposed model is less likely to miss the influential conditional dependency among genes. While CAPME and JML score near 1 in sensitivity, they mark poor number in specificity because 5-fold cross-validation for CAPME and validation approach for JML with default setting for tuning parameters selection tend to choose small ones, leading to dense estimates. JMCCM produces higher Youden's index across all simulation scenarios compared to all other methods, and the performance gap between JMCCM and others is increasingly widened as the models increase in sample size and complexity. The results for the large-scale simulations (models 7–9) are summarized in Table 4 and Table S3. Due to long computing time, the results of CAPME for models 8 and 9 are not reported. Overall, the results are consistent with the small-scale simulations. The gap between JMCCM and SCGGM in estimation performance widened as the complexity of the model increases (Table 4).

We also simulate SNPs to mimic the linkage disequilibrium (LD) which is known to be a common phenomenon in DNA sequence and to assess the performance of our approach. We randomly generate two groups of SNPs which lie on LD, each of which contains LD block including 10 correlated SNPs such that correlation is greater than 0.9. Together with these SNPs, 10 SNPs are generated independently from Bernoulli trials with probability of 0.5 for a total of 20 SNPs and 20 genes: *m* = 20 and *p* = 20.  Table 5 presents the simulation results using these data of sample size *N* = 500 and *N* = 1000 with 50 repetitions. Compared to the results of model 4 in Table 3, which is based on 20 independent SNPs with *N* = 500, LD is found to have little impact on all methods, except for slight decreases in Stein loss and Frobenius norm of SCGGM.

## 5. eQTL Analysis of Yeast Data

In this section, we apply the proposed algorithm to genomic eQTL (i.e., expression quantitative trait loci) data in order to examine whether the proposed method effectively recovers true dependency of gene expressions, which builds on known molecular mechanisms. To this end, we collect a set of yeast data [22], including polymorphic genotypes and mRNA expressions. The yeast data have been widely applied to elucidate the biological interactions between nucleotide polymorphisms and their responding genes (e.g., perturbation effects) [23–26]. Thus, our primary goal is to identify conditional dependency among genes with an adjustment of SNPs perturbations to each gene expression level.

The data sets are collected for two yeast parent strains, BY4716 (BY) and RM11-1a (RM), and their 112 segregants. We obtain SNPs for 1,260 loci to the exclusion of the redundant SNPs observed in neighboring genetic regions and leave 3,684 expression genes after screening out genes of missing more than 5%. In order to validate whether or not the estimated gene network is consistent with unknown biological knowledge, we take the true signaling pathway for comparison. Out of 3,684 yeast genes, we purposely focus on a set of 64 genes that are ascertained in the cell cycle yeast pathway available in the KEGG database [24]. Together with 1,260 SNPs as predictors, we construct a gene network model of 64 genes. JMCCM selects 222 nonzero elements of the precision matrix and leaves nonzero regression coefficients, for a total of 111 links among genes. A total of 489 regression coefficients of SNPs over gene expression levels are included in the final model.  Figure 2 displays the conditional gene network estimated by the proposed joint model. A pair of two genes is linked if an off-diagonal element of the estimated precision matrix is nonzero, and so 51 genes are shown to be linked and assemble in a module.

**Figure 1:**
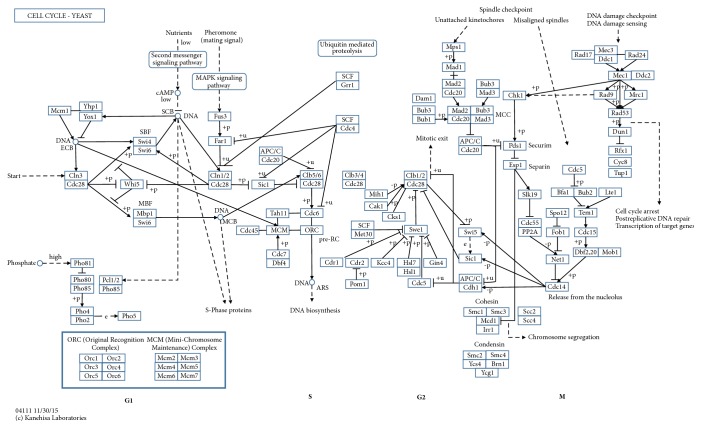
The yeast cell cycle pathway from the KEGG database. Source: http://www.kegg.jp/kegg-bin/highlight_pathway?scale=1.0&map=map04111&keyword=cell%20cycle.

**Figure 2:**
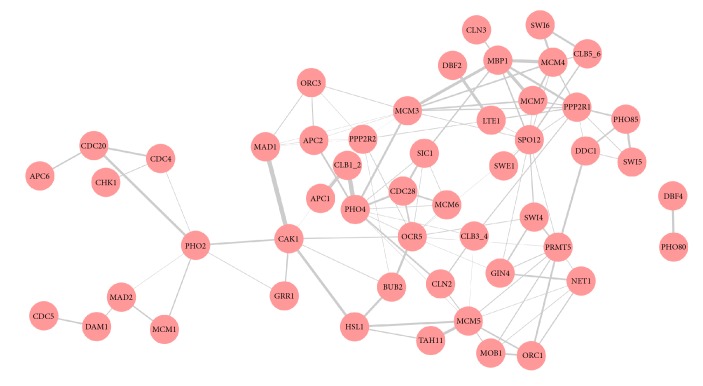
The gene network of the yeast data related to the cell cycle pathway via the JMCCM.

Given this estimated gene network, we notice that the gene network is found to be concordant with the true cell cycle pathway. For instance, according to the true cell cycle pathway in Figure 1, MCM3 is linked to ORC3, ORC5, MCM7, and MCM4. MCM5 is connected to TAH11 and ORC1. PHO4 is closely linked to CLN2 and SIC1, whose molecular function is related to MAP kinase orthologs (i.e., MAPK pathway genes). It is interesting to note that CLN3 and SWI4 are mutually connected together, linking to their downstream gene MBP1. Hence, this undirected graph contributes to recovering lots of links among the 64 genes of the pathway.

With regard to genetic variant effects, JMCCM is shown to effectively identify some of the well-known direct genetic perturbations. Gene expressions are regulated by some genetic variants, which, unless otherwise taken into account, may falsely capture the interplay of genes in a network. Our founding includes Clb-specific Cdk inhibitor (SIC1) as influencing the molecular interface between cyclin-Cdk complexes (e.g., binding to and blocking Cdk1/Clb activity, ultimately to maneuver the timing of DNA replication (see Table S1 in Supplementary Materials; [27]). In addition, our gene networks demonstrate that SIC1 is strongly perturbed by CLB3, while SCGGM did not detect any perturbation effects. More interestingly, many previous experiments validated the association between SIC1 and CLB3 to uncover the underlying mechanism of the cell cycle [28–30]. Clearly, this implies that JMCCM does a better job in accounting for SNPs perturbation as compared to SCGGM.

Moreover, the gene module detected from JMCCM is shown in Table 6. Pertaining to these gene modules, we hypothesize if gene modules, each containing hub genes and their neighboring genes, are enriched with common biological processes or not. To test this hypothesis, we conduct the gene ontology (GO) enrichment analysis [31] over the detected gene network modules (see Table S2 in Supplementary Materials) from both JMCCM and SCGGM using Fisher's exact tests. Table 6 demonstrates that the proposed method outstandingly performs detecting modules biologically associated with many molecular processes, while none is detected by SCGGM. More importantly, the pathway of the organic acid metabolic process enriched in gene module 1 is also reported and validated on the basis of the network modules constructed with large-scale integration of yeast data in Zhu* et al.* [32].

Putting all things together, we conclude that the proposed method facilitates recovering the true SNPs perturbations in the midst of gene regulations and elucidating the underlying interplay of gene interactions. These fortes of the proposed algorithm are favored for reinforcing* a priori* biological knowledge and address a novel hypothesis related to clinical and translational potential.

## 6. Conclusion and Discussion

In this paper, we propose JMCCM to efficiently identify conditional dependent structures of gene expressions with adjustments to perturbation effects of SNPs. Contrary to the existing conditional graphical models, the precision matrix commonly used to reveal the true relationship among genes is parameterized via the modified Cholesky decomposition. The maximum likelihood estimates of the precision matrix were computed, while variable selection of SNPs and Cholesky factors are carried out separately and jointly by the GCV and BIC criterion. From experimental studies, it is clearly shown that JMCCM performs better than the existing penalization methods. Besides, JMCCM in the application to yeast cell cycle data successfully recovers many parts of the cell cycle pathway with adjustments of SNPs to each gene expressions level. Notably, the model entails the estimation of precision matrix, of which components are assumed to be constant. So, in the future, we may relax this somewhat strong assumption in the way that the model can parametrize over *τ* and *ϕ* in pursuit of more accurate estimation [13]. We leave this for next study.

## Figures and Tables

**Algorithm 1:**
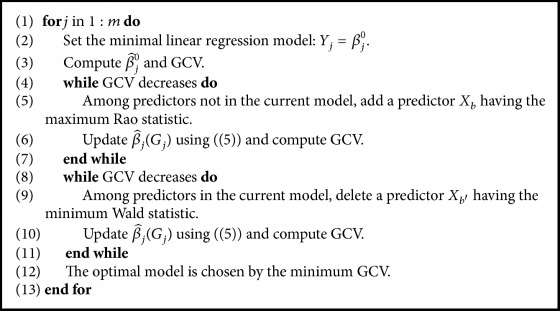
Variable selection in the mean vector estimation.

**Algorithm 2:**
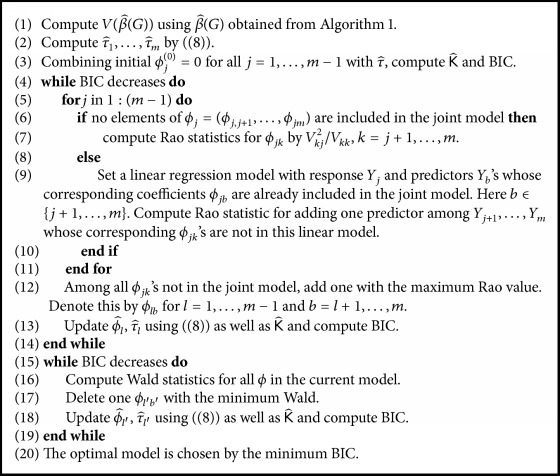
Variable selection in the precision matrix estimation.

**Algorithm 3:**
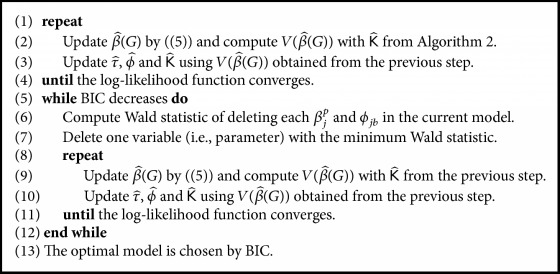
Variable selection in the joint estimation.

**Table 1:** Six scenarios for small-scale experimental study.

Model	m	p	*ℙ*(*k*_*ij*_ ≠ 0)	*ℙ*(*β*_*j*_^*l*^ ≠ 0)
1	10	10	2/*m*	3.5/*p*
2	20	10	2/*m*	3.5/*p*
3	40	10	2/*m*	3.5/*p*
4	20	20	2/*m*	4/*p*
5	30	30	2/*m*	4/*p*
6	40	40	2/*m*	4/*p*

**Table 2:** Three scenarios for large-scale experimental study.

Model	m	p	*ℙ*(*k*_*ij*_ ≠ 0)	*ℙ*(*β*_*j*_^*l*^ ≠ 0)
7	100	100	2/*m*	3/*p*
8	200	200	2.5/*m*	15/*p*
9	400	200	1.5/*m*	20/*p*

**Table 3:** Comparisons of the performance of JMCCM with SCGGM, CAPME, and JML for models 1–6. Standard errors are presented in parenthesis.

Model	Method	*δ*_*Stein*_	‖Δ‖_*F*_	SPE	SEN	Youden
*N* = 500						
1	JMCCM	0.139 (0.004)	0.519 (0.008)	0.864 (0.010)	0.723 (0.014)	0.587 (0.012)
	SCGGM	0.171 (0.007)	0.556 (0.010)	0.865 (0.030)	0.624 (0.027)	0.506 (0.014)
	CAPME	0.113 (0.003)	0.512 (0.008)	0.002 (0.001)	1.000 (0.000)	0.002 (0.001)
	JML	0.099 (0.003)	0.450 (0.005)	0.160 (0.026)	0.980 (0.005)	0.140 (0.024)

*N* = 500						
2	JMCCM	0.241 (0.005)	0.677 (0.009)	0.915 (0.004)	0.801 (0.007)	0.716 (0.006)
	SCGGM	0.349 (0.008)	0.821 (0.012)	0.661 (0.029)	0.882 (0.016)	0.542 (0.017)
	CAPME	0.430 (0.007)	1.028 (0.011)	0.005 (0.001)	1.000 (0.000)	0.005 (0.001)
	JML	0.232 (0.004)	0.670 (0.008)	0.210 (0.009)	0.980 (0.003)	0.200 (0.009)

*N* = 500						
3	JMCCM	0.705 (0.011)	1.145 (0.009)	0.947 (0.002)	0.653 (0.006)	0.600 (0.005)
	SCGGM	1.158 (0.034)	1.506 (0.032)	0.716 (0.036)	0.706 (0.029)	0.422 (0.010)
	CAPME	1.703 (0.012)	2.217 (0.015)	0.005 (0.000)	1.000 (0.000)	0.005 (0.000)
	JML	-	-	-	-	-

*N* = 500						
4	JMCCM	0.239 (0.006)	0.678 (0.011)	0.910 (0.004)	0.802 (0.007)	0.712 (0.007)
	SCGGM	0.402 (0.010)	0.869 (0.011)	0.825 (0.031)	0.772 (0.017)	0.597 (0.019)
	CAPME	0.447 (0.007)	1.081 (0.010)	0.004 (0.001)	1.000 (0.000)	0.004 (0.001)
	JML	0.284 (0.005)	0.793 (0.008)	0.110 (0.006)	1.000 (0.001)	0.100 (0.006)

*N* = 500						
5	JMCCM	0.463 (0.007)	0.953 (0.007)	0.941 (0.003)	0.731 (0.007)	0.672 (0.006)
	SCGGM	0.743 (0.011)	1.188 (0.010)	0.978 (0.002)	0.567 (0.007)	0.545 (0.007)
	CAPME	0.983 (0.008)	1.683 (0.012)	0.005 (0.001)	1.000 (0.000)	0.005 (0.001)
	JML	0.524 (0.524)	1.074 (0.009)	0.140 (0.006)	0.980 (0.002)	0.120 (0.006)

*N* = 500						
6	JMCCM	0.711 (0.010)	1.153 (0.010)	0.951 (0.002)	0.639 (0.006)	0.590 (0.005)
	SCGGM	1.126 (0.011)	1.438 (0.007)	0.988 (0.001)	0.440 (0.004)	0.428 (0.004)
	CAPME	1.781 (0.013)	2.406 (0.013)	0.006 (0.000)	0.999 (0.000)	0.006 (0.000)
	JML	-	-	-	-	-

*N* = 1000						
1	JMCCM	0.068 (0.002)	0.364 (0.006)	0.814 (0.007)	0.886 (0.009)	0.700 (0.009)
	SCGGM	0.139 (0.010)	0.483 (0.018)	0.888 (0.024)	0.668 (0.031)	0.556 (0.016)
	CAPME	0.055 (0.002)	0.346 (0.006)	0.003 (0.001)	1.000 (0.000)	0.003 (0.001)
	JML	0.046 (0.001)	0.301 (0.005)	0.150 (0.015)	1.000 (0.001)	0.150 (0.015)

*N* = 1000						
2	JMCCM	0.117 (0.003)	0.466 (0.006)	0.894 (0.003)	0.898 (0.005)	0.793 (0.004)
	SCGGM	0.168 (0.007)	0.550 (0.010)	0.652 (0.018)	0.960 (0.006)	0.612 (0.016)
	CAPME	0.215 (0.003)	0.696 (0.007)	0.003 (0.001)	1.000 (0.000)	0.003 (0.001)
	JML	0.119 (0.002)	0.459 (0.006)	0.320 (0.120)	0.990 (0.003)	0.310 (0.012)

*N* = 1000						
3	JMCCM	0.321 (0.006)	0.766 (0.007)	0.916 (0.002)	0.808 (0.004)	0.724 (0.003)
	SCGGM	0.645 (0.015)	1.107 (0.011)	0.674 (0.021)	0.847 (0.024)	0.521 (0.008)
	CAPME	0.824 (0.006)	1.405 (0.007)	0.005 (0.000)	1.000 (0.000)	0.005 (0.000)
	JML	-	-	-	-	-

*N* = 1000						
4	JMCCM	0.115 (0.003)	0.467 (0.006)	0.893 (0.003)	0.904 (0.006)	0.797 (0.005)
	SCGGM	0.286 (0.009)	0.745 (0.012)	0.786 (0.028)	0.822 (0.019)	0.609 (0.015)
	CAPME	0.215 (0.003)	0.703 (0.006)	0.003 (0.001)	1.000 (0.000)	0.003 (0.001)
	JML	0.123 (0.002)	0.486 (0.005)	0.160 (0.006)	1.000 (0.002)	0.150 (0.005)

*N* = 1000						
5	JMCCM	0.217 (0.004)	0.643 (0.006)	0.924 (0.002)	0.867 (0.005)	0.792 (0.004)
	SCGGM	0.671 (0.011)	1.141 (0.001)	0.995 (0.002)	0.542 (0.009)	0.536 (0.008)
	CAPME	0.473 (0.004)	1.065 (0.007)	0.004 (0.000)	1.000 (0.000)	0.004 (0.000)
	JML	-	-	-	-	-

*N* = 1000						
6	JMCCM	0.331 (0.005)	0.781 (0.006)	0.926 (0.002)	0.789 (0.005)	0.715 (0.004)
	SCGGM	1.024 (0.010)	1.380 (0.006)	0.998 (0.000)	0.414 (0.003)	0.412 (0.003)
	CAPME	0.844 (0.006)	1.466 (0.008)	0.005 (0.000)	0.999 (0.000)	0.004 (0.000)
	JML	-	-	-	-	-

**Table 4:** Comparisons of the performance of JMCCM with SCGGM and CAPME for models 7–9. Standard errors are presented in parenthesis.

Model	Method	*δ*_*Stein*_	‖Δ‖_*F*_	SPE	SEN	Youden
*N* = 500						
7	JMCCM	2.234 (0.021)	2.071 (0.011)	0.969 (0.001)	0.638 (0.004)	0.608 (0.003)
	SCGGM	2.670 (0.017)	2.160 (0.007)	0.994 (0.000)	0.512 (0.002)	0.506 (0.002)
	CAPME	12.914 (0.038)	9.257 (0.028)	0.002 (0.000)	1.000 (0.000)	0.002 (0.000)

*N* = 500						
8	JMCCM	6.560 (0.073)	3.669 (0.028)	0.982 (0.001)	0.475 (0.004)	0.457 (0.003)
	SCGGM	10.687 (0.035)	4.318 (0.006)	0.925 (0.000)	0.430 (0.002)	0.355 (0.002)
	CAPME	-	-	-	-	-

*N* = 500						
9	JMCCM	16.703 (0.254)	5.998 (0.062)	0.986 (0.001)	0.466 (0.003)	0.452 (0.002)
	SCGGM	34.025 (0.722)	7.277 (0.048)	0.874 (0.005)	0.453 (0.003)	0.328 (0.003)
	CAPME	-	-	-	-	-

**Table 5:** Comparisons of the performance of JMCCM with SCGGM and CAPME for the experiment with highly correlated SNPs. 20 genes (m) and 20 SNPs (p) are involved. Standard errors are presented in parenthesis.

Method	*δ*_*Stein*_	‖Δ‖_*F*_	SPE	SEN	Youden
*N* = 500					
JMCCM	0.239 (0.006)	0.678 (0.011)	0.910 (0.004)	0.802 (0.007)	0.712 (0.007)
SCGGM	0.402 (0.010)	0.869 (0.011)	0.825 (0.031)	0.772 (0.017)	0.597 (0.019)
CAPME	0.447 (0.007)	1.081 (0.010)	0.004 (0.001)	1.000 (0.000)	0.0004 (0.001)

*N* = 1000					
JMCCM	0.115 (0.003)	0.467 (0.006)	0.893 (0.003)	0.904 (0.006)	0.797 (0.005)
SCGGM	0.286 (0.009)	0.745 (0.012)	0.786 (0.028)	0.822 (0.019)	0.609 (0.015)
CAPME	0.215 (0.003)	0.703 (0.006)	0.003 (0.001)	1.000 (0.000)	0.003 (0.001)

**Table 6:** Gene ontology (GO) enrichment analysis over the genes in detected module from the JMCCM.

Module	Module size	GO category	GO enrichment *p* value
Module 1	94	Carboxylic acid metabolic process	0.00101
		Carboxypeptidase activity	0.00904
		Catabolic process	0.00101
		Cellular catabolic process	0.00101
		Exopeptidase activity	0.00904
		Metalloexopeptidase activity	0.00904
		Metallopeptidase activity	0.00904
		Organic acid metabolic process	0.00101
		Peptidase activity	0.00904

## Data Availability

The source of the eQTL data analyzed in this paper is [22]. An R package JMCCM and the eQTL data set are available at author's webpage: https://sites.google.com/site/sunghwanshome/.

## Appendix

### A. Positive Definiteness of *K*

The matrix representation of the modified Cholesky decomposition (2) is given by

(A.1) Cϕ=1−ϕ12…−ϕ1m1…−ϕ2m⋱⋮1,Dτ=τ1τ2⋱τm.

For simplicity, write *C* = *C*(*ϕ*) and *D* = *D*(*τ*). For any nonzero vector *u* ∈ *ℝ*^*m*^,

(A.2) u⊤Ku=u⊤C⊤DCu=Cu⊤DCu=∑j=1mCuj2τj>0.

Since *τ*_*j*_ > 0 for all *j* = 1,…, *m*, ∑_*j*=1_^*m*^((*Cu*)_*j*_)^2^*τ*_*j*_ = 0 if and only if *Cu* = 0, which cannot happen here because *u* is a nonzero vector. Thus *K* is a positive definite matrix.

### B. A Derivation of the Log-Likelihood Function

The probability density function of (1) is given by

(B.1) fy ∣ x;θ=K1/22πm/2exp−12y−μx⊤Ky−μx,

where *θ* = (*β*, *ϕ*, *τ*). The log-likelihood function corresponding to JMCCM can be derived as follows:

(B.2) lθ=log∑i=1Nfyi ∣ xi;θ=−mN2log2π+N2logK−12∑i=1Nyi−μi⊤Kyi−μi=N2−mlog⁡2π+∑j=1mlog⁡τj−trKVβ

where the last equality comes from |*C*| = |*C*^*⊤*^| = 1, |*D*| = *τ*_1_*τ*_2_ ⋯ *τ*_*m*_, and

(B.3) $$\begin{array}{c} \log\left|\;K\;\right|=log\left|\;C^{⊤}\;\right|\left|\;D\;\right|\left|\;C\;\right|=\log\left|\;D\;\right|=\overset{m}{\underset{j=1}{\sum }}\log\tau_{j}. \end{array}$$

### C. Derivations of MLE $\hat{\beta }$, $\hat{\phi }$, and $\hat{\tau }$

Using notations presented in Section 2.2, optimization problem for $\hat{\beta }(G)$ is expressed as

(C.1) max12log⁡K−Y−XβG⊤WY−XβG.

Denote the score function of *β*(*G*) by *S*(*β*(*G*)). Observe that

(C.2) SβG=∂∂βG−12Y⊤WY−2βG⊤X⊤WY+βG⊤X⊤WXβG−∑j=1mlog⁡τj=X⊤WY−X⊤WXβG

By solving the normal equation *S*(*β*(*G*)) = 0, we have (5).

To derive (6), let *C*(*ϕ*_*j*_) = (0,…, 0,1, *ϕ*_*j*,*j*+1_,…, *ϕ*_*jm*_), *j*th row of *C*. Then we can see that *C*^*⊤*^*DC* = ∑_*j*=1_^*m*^*τ*_*j*_*C*(*ϕ*_*j*_)*C*(*ϕ*_*j*_)^*⊤*^. The log-likelihood function (3) can be expressed as the sum of *ℓ*_*j*_(*θ*_*j*_) as follows:

(C.3) lθ=N2−log2π+∑j=1mlog⁡τj−trKVβ=N2−mlog⁡2π+∑j=1mlog⁡τj−tr∑j=1mτjCϕjCϕj⊤Vβ=N2−mlog⁡2π+∑j=1mlog⁡τj−∑j=1mτjCϕj⊤VβCϕj=N2−mlog⁡2π+∑j=1mlog⁡τj−∑j=1mτjVjj−2ϕj⊤Vj,21+ϕj⊤Vj,22ϕj=∑j=1mljθj.

Finally, for any given *j* = 1,…, *m* − 1, the gradient of log-likelihood function with respect to *ϕ*_*j*_ and *τ*_*j*_ is given by

(C.4) ∇ljθj=NτjVj,21−Vj,22ϕjN21τj−Vjj−2ϕj⊤Vj,21+ϕj⊤Vj,22ϕj.

Then (8) is obtained by solving ∇*ℓ*_*j*_(*θ*_*j*_) = 0 with respect to *ϕ*_*j*_ and *τ*_*j*_.

## Supplementary Materials

Supplementary Materials Table S1 shows the estimated coefficient matrix from JMCCM applied to the genes of cell cycle pathway. The columns refer to genes and the rows to SNPs. Table S2 contains the gene lists of each module estimated from both SCGGM and JMCCM. Table S3 shows results of simulation with a large data set of m = 1000 (genes), p = 4000 (SNPs), and N = 500.
